# Differences in inflammatory marker profiles and cognitive functioning between deficit and nondeficit schizophrenia

**DOI:** 10.3389/fimmu.2022.958972

**Published:** 2022-10-19

**Authors:** Dandan Wang, Yewei Wang, Yan Chen, Lingfang Yu, Zenan Wu, Ruimei Liu, Juanjuan Ren, Xinyu Fang, Chen Zhang

**Affiliations:** ^1^ Schizophrenia Program, Shanghai Mental Health Center, Shanghai Jiao Tong University School of Medicine, Shanghai, China; ^2^ The Affiliated Brain Hospital of Nanjing Medical University, Nanjing, China

**Keywords:** schizophrenia, deficit syndrome, cognitive function, C-reactive protein, cytokine, inflammation

## Abstract

Deficit schizophrenia (DS) patient is a homogenous subtype of schizophrenia that includes primary and enduring negative symptoms. This study aimed to compare the differences in cognitive functioning and plasma levels of C-reactive protein (CRP) and inflammatory cytokines among DS patients, nondeficit schizophrenia (NDS) patients, and healthy controls (HCs). A total of 141 schizophrenia patients and 67 HCs were included in this study. The schizophrenia patients were divided into DS (N= 51) and NDS (N=90) groups based on the Proxy for the Deficit Syndrome Scale (PDS). The Positive and Negative Syndrome Scale (PANSS) and the Repeatable Battery for the Assessment of Neuropsychological Status (RBANS) were used to evaluate the clinical symptoms and cognitive performances, respectively. The plasma level of CRP, IL-1β, Il-2, IL-4, IL-6, IL-8, IL-10, IL-12, IL-17, TNF-α, and IFN-γ were measured using enzyme-linked immunosorbent assays (ELISAs). Our results showed that DS patients had the worst cognitive performance, especially in the immediate memory, attention, and language dimensions, compared to the NDS and HC groups. Compared to the HCs group, DS patients had higher levels of CRP, IL-1β, IL-6, IL-8, IFN-γ, and total proinflammatory cytokines, and NDS patients had higher levels of IL-1β, IFN-γ, and proinflammatory cytokines. We also found that CRP levels were significantly increased in DS patients compared to NDS patients. Moreover, stepwise logistic regression analysis revealed that CRP is an independent risk factor for DS. Sex stratification analysis showed significant differences in almost all cytokines in female samples but not in male samples. The significant differences in cognitive performance and inflammatory components among groups suggest that deficit syndrome is an independent endophenotype of schizophrenia patients with unique immune-inflammatory features, but may have sex characteristics.

## Introduction

Schizophrenia is a chronic and debilitating disease that affects approximately 0.75% of the Chinese population and places a heavy burden on suffers and families (1). It is characterized by disturbances in perception, thinking, emotions, cognition, and behaviors, and always results in poor social functions. Clinically, the considerable heterogeneity of disease manifestations presents a huge challenge to understanding the underlying neurobiological mechanism of this disease. Therefore, researchers propose to categorize schizophrenia patients into different subtypes and subpopulations; this is, a practical approach to minimize the heterogeneity of schizophrenia. Hughlings Jackson provided one of the first methods to divide the symptomatology of schizophrenia into positive and negative symptoms (2). However, there is still a great deal of heterogeneity even within these categories. Deficit syndrome in schizophrenia, which is defined by the presence of primary symptoms (independent of treatment and depressive symptoms, or not due to positive symptoms) and enduring negative symptoms, is thought to be a more homogeneous manifestation of schizophrenia (3). Subsequent studies have shown that classification using deficit syndrome has good confidence and high stability (Amador et al., 1999; Kirkpatrick et al., 1994). Consequently, the study of this more homogeneous subgroup may enhance the understanding of the prognosis of schizophrenia, help to reveal valuable biomarkers for schizophrenia, and to promote precision treatment and intervention strategies.

A recent meta-analysis showed that deficit schizophrenia (DS) patients account for one-third of all schizophrenia patients (López-Díaz et al., 2018). Evidence has shown that DS patients have significant differences in sociodemographic and clinical characteristics compared to nondeficit schizophrenia (NDS) patients. Previous studies indicated that the male sex, with a family history, and a lower prevalence of cannabis use were risk factors for DS (4, 5). Although an increasing amount of evidence supports that winter birth is strongly associated with an excess of schizophrenia (6), a pooled analysis from 6 countries demonstrated that summer birth significantly increased the risk of deficit schizophrenia (7). For clinical characteristics, a recent investigation conducted in China indicated that DS patients had significantly more severe negative symptoms, but lower scores on positive symptoms, general psychopathology, and depressive symptoms than NDS patients (8). Furthermore, DS patients have been observed to have different levels of cognitive functioning compared with NDS patients, with some studies reporting impairments in global and extensive cognitive functions among the former group (9) and other studies finding impairments only in specific domains of cognition such as working memory, visual-spatial memory, executive function and verbal fluency (10–12). Hence, the relationship between cognitive function and deficit syndrome in schizophrenia requires more investigation. Taken together, the above differences between DS and NDS patients all suggest that DS is a unique subtype of schizophrenia and may have specific pathophysiological mechanisms.

Contemporary evidence supports the hypothesis of perturbed inflammatory processes in the pathogenesis of schizophrenia, as clinical and preclinical studies both showed that prenatal infection or maternal immune alterations during pregnancy significantly increased the risk of schizophrenia in offspring (13, 14). To date, many studies have suggested a link between peripheral inflammatory biomarkers and the emergence of negative symptoms in schizophrenia in particular, with dysregulation of immune cells and aberrations in proinflammatory cytokines noted as important (15, 16). Therefore, DS patients, with primary and enduring negative as the main manifestations, may also have a specific immune-inflammatory state. However, only a few studies have focused on this homogeneous subtype of schizophrenia patients to identify its unique immune-inflammatory characteristics. For instance, Garcia-Rizo et al. found that DS patients (N=20) had higher concentrations of C-reactive protein (CRP) and interleukin-6 (IL-6) than NDS patients (N=42) (17), while another study found that tumor necrosis factor-α (TNF-α) and IL-6 levels were elevated in DS patients (N=17) compared to NDS patients (N=39). Interestingly, a recent study with 54 schizophrenia patients found only an increased level of IL-17 in DS patients, but no differences in IL-1β, TNF-α, IL-12, IL-10, interferon-γ (IFN-γ), or transforming growth factor-β (TGF-β) were observed (18). Since all these studies had small sample sizes and only limited inflammatory cytokines were measured, further research is warranted.

In the present study, we recruited a relatively larger sample of patients with schizophrenia and measured more plasma inflammatory cytokines, including IL-1β, IL-2, IL-4, IL-6, IL-8, IL-10, IL-12, IL-17, TNF-α, IFN-γ, and CRP. We aimed to 1) compare the clinical characteristics, especially cognitive functions among DS, NDS patients, and healthy controls (HCs), and 2) compare the plasma immune-inflammatory cytokines among DS patients, NDS patients, and the HCs group, and to identify distinct patterns of immune inflammation in DS patients. Specifically, we hypothesized that DS patients have more extensive and severe cognitive impairments than NDS patients, as well as unique immune-inflammatory changes.

## Methods

### Participants

In this study, 141 outpatients with schizophrenia were recruited from Shanghai Mental Health Center. Each patient was diagnosed with schizophrenia in strict accordance with the structured clinical interview standard of DSM-5. The detailed patient inclusion criteria are as follows: 1) aged 18–60 years; 2) the course of the disease is more than 1 year; 3) junior high school or above education level; and 4) Chinese Han population. Patients were excluded if they met the following criteria: 1) received electroconvulsive therapy (ECT) within six months; 2) comorbidity with mental disorders caused by organic diseases, mental disorders caused by drugs or alcohol, and other mental disorders; 3) comorbidity with serious physical diseases and autoimmune diseases; 4) patients with infection, fever or use of drugs that affect the immune-inflammatory system within the last month; 5) pregnant or breastfeeding women; or 6) alcohol or substance abuse. Additionally, 67 HCs were matched with schizophrenia patients based on general demographic data and were recruited by word of mouth from the same catchment area as the patients. None of the healthy subjects presented with a personal or family history of mental disorders had fever, infection, or received any drugs that affect the immune-inflammatory system within the last month.

This study was approved by the Medical Ethics Committee of Shanghai Mental Health Center. All participants signed informed consent forms before performing all procedures related to this study. This study was conducted in strict conformity with the Declaration of Helsinki and other relevant national and international regulations.

### Clinical assessments

The participants were administered the Structured Clinical Interview for DSM-V (SCID) by experienced psychiatrists. Additionally, psychiatrists collected demographic information from the participants and compiled a narrative case summary. Clinical symptoms of patients were assessed using the Positive and Negative Syndrome Scale (PANSS). To ensure the reliability and consistency of the overall study assessment, two psychiatrists with at least five years of work experience were trained in the PANSS before the study. After training, the interobserver correlation coefficient for the PANSS score remained above 0.8.

The proxy for Deficit Syndrome (PDS) was used to classify patients into the DS or NDS group. The PDS score is defined as the sum of the scores of the anxiety, guilt feelings, depressive mood, and hostility items subtracted from the score for blunted affect (19). The validity and stability of the PDS for distinguishing between DS and NDS have previously been demonstrated (19–21). Finally, the patients were divided into 51 cases of DS and 90 cases of NDS.

Cognitive function in all participants was assessed by the Repeatable Battery for the Assessment of Neuropsychological Status (RBANS) (22), which evaluates 5 dimensions of neurocognition: immediate memory, visuospatial/constructional, language, attention, and delayed memory. It works well in cognitive assessment and exhibits good reliability and validity in the general population and schizophrenia patients (23, 24).

### Plasma cytokines measurement

Blood samples were drawn from all participants in the fasting state between 7 and 9 am on the day of enrollment. Plasma CRP, IL-1β, IL-2, IL-4, IL-6, IL-8, IL-10, IL-12, IL-17, TNF-α, and IFN-γ were measured using ELISA kits for humans according to the manufacturer’s instructions (Jiangsu Meimian Industrial Co., Ltd).

### Data analysis

The Statistical Package for the Social Sciences (SPSS) version 23.0 was used for data analysis. Categorical variables were analyzed by the chi-square test, and continuous variables were analyzed by two-sample t tests or one-way analysis of variance (ANOVA). The normality test for inflammatory factors was performed using the Shapiro–Wilk test. Since all plasma cytokines had nonnormal distributions, the Kruskal–Wallis test was used to compare the differences in cytokine levels among the three groups. Bonferroni correction was performed for multiple tests. In addition, we performed sex stratification analysis of groups in various cytokines. The correlations between variables were conducted by partial correlation analysis. Univariate logistic regression analysis was used to detect factors that affected the classification of patients. Finally, stepwise logistic regression analysis was performed to explore the model dividing patients into DS or NDS. ROC curves were utilized to prove the possibility that plasma cytokines can be used as potential biomarkers to differentiate between patients with DS and NDS. For all analyses, the results were considered statistically significant at p < 0.05 with a two-tailed test.

## Results

### Sociodemographic and clinical characteristics among DS, NDS, and HC groups

Of the 141 patients with schizophrenia, 51 patients (36.17%) were classified as the DS subtype. **Table 1** shows the basic demographic and clinical characteristics of DS patients, NDS patients, and HCs. There were no significant differences among the DS, NDS, and HC groups in terms of age, sex, or education (all P > 0.05). We also found no significant differences in age of onset, total disease course, family history, or olanzapine equivalents between DS and NDS patients (all P > 0.05). Compared with NDS patients, patients with DS had more severe negative symptoms (t = 4.681, P < 0.001), but fewer positive symptoms (t = 3.728, P < 0.001). There were no significant differences in general psychopathology or PANSS total scores between the two patient groups (all p > 0.05). Our results showed that both DS and NDS patients had significantly poorer cognitive performance than the HC group (all P < 0.01). *Post hoc* test with LSD showed that, compared to NDS patients, DS patients presented lower scores in RBANS total scale (P_LSD_ = 0.011) and on the immediate memory (P_LSD_ = 0.007), attention (P_LSD_ = 0.032) and language (P_LSD_ = 0.023) dimensions compared to NDS patients.

**Table 1:** Demographics, and cognitive function in deficit and nondeficit schizophrenia.

	DS (N =5 1)	NDS (N = 90)	HCs (N = 67)	t/F/X^2^	P
Age (year)	29.92 ± 9.27	31.94 ± 9.30	29.43 ± 6.38	1.934	0.147
Sex (male/female)	24/27	34/56	30/37	1.396	0.498
BMI (kg/m^2^)	22.34± 3.53	22.17 ± 3.49	21.86 ± 2.72	0.330	0.719
Education (year)	12.47 ± 2.84	13.53 ± 2.95	13.60 ± 3.21	2.535	0.082
Age of onset (year)	27.96 ± 7.61	27.92 ± 7.90		0.033	0.974
Total disease course (year)	3.70 ± 3.70	4.63 ± 4.49		1.257	0.211
Family history (no/yes)	11/31	23/43		0.041	0.839
Olanzapine equivalents	11.13 ± 7.00	9.45 ± 6.67		1.064	0.292
PANSS					
Positive subscale	15.61 ± 6.00	19.78 ± 6.59		3.728	< 0.001*
Negative subscale	22.80 ± 7.31	16.94 ± 7.05		4.681	< 0.001*
General subscale	38.55 ± 12.68	40.10 ± 11.52		0.755	0.452
Total score	76.69 ± 22.26	76.82 ± 22.16		0.034	0.973
RBANS					
Immediate memory^1^	62.59 ± 15.59	70.73 ± 18.23	92.18 ± 12.13	50.608	< 0.001*
Visuospatial constructional	84.55 ± 16.47	86.06 ± 15.61	101.25 ± 15.33	22.860	< 0.001*
Attention^2^	85.17 ± 16.65	91.03 ± 16.43	114.22 ± 13.27	62.775	< 0.001*
Language^3^	73.84 ± 14.91	79.83 ± 14.79	96.15 ± 14.98	37.680	< 0.001*
Delayed memory	69.75 ± 20.50	74.61 ± 17.48	96.31 ± 12.99	44.491	< 0.001*
Total score^4^	68.59 ± 14.90	75.11 ± 14.67	99.84 ± 13.99	82.396	< 0.001*

DS, Deficit schizophrenia; NDS, Nondeficit schizophrenia; HCs, Healthy controls. Data were presented in Mean ± SD.

DS vs NDS:^1^LSD corrected P = 0.007; ^2^LSD corrected P = 0.032; ^3^LSD corrected P = 0.023; ^4^LSD corrected P = 0.011.

DS vs HC:^1^LSD corrected P < 0.001; ^2^LSD corrected P < 0.001; ^3^LSD corrected P < 0.001; ^4^LSD corrected P < 0.001.

NDS vs HC:^1^LSD corrected P < 0.001; ^2^LSD corrected P < 0.001; ^3^LSD corrected P < 0.001; ^4^LSD corrected P < 0.001. *P < 0.05.

### Plasma cytokines in DS, NDS, and HCs

The sum of IL-1β, IL-2, IL-6, IL-8, IL-12, IL-17, TNF-α, and IFN-γ was regarded as the level of proinflammatory cytokines, whereas the sum of levels of IL-4 and IL-10 was regarded as the level of anti-inflammatory cytokines.

**Figure 1:**
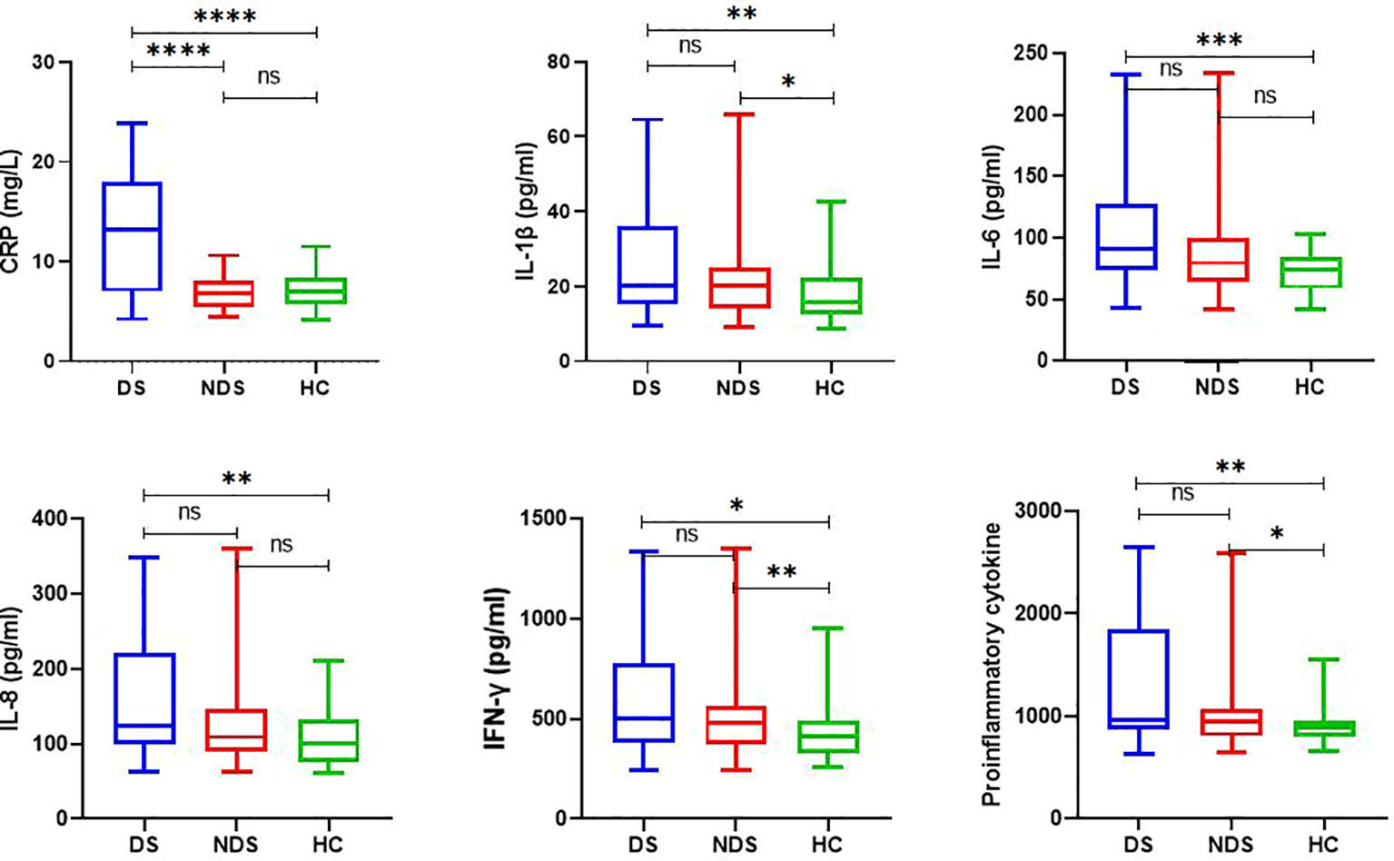
Plasma levels of inflammatory cytokines in control subjects, deficit and nondeficit schizophrenia patients. Each bar represents the median level of cytokines. Error bars represent the interquartile. DS, Deficit schizophrenia; NDS, Nondeficit schizophrenia; HC, Healthy controls. (*p < 0.05, **p < 0.001, ***p < 0.0005, ****p < 0.0001). ns =No statistical difference.

As shown in **Table 2** and **Figure 1**, the plasma levels of CRP (Z = 37.764, P < 0.001), IL-1β (Z = 10.554, P = 0.004), IL-6 (Z = 14.681, P = 0.001), IL-8 (Z = 12.583, P = 0.002), IFN- γ (Z = 11.511, P = 0.003), and proinflammatory cytokines (Z = 11.80, P = 0.003) varied significantly among DS patients, NDS patients, and HCs. After the Bonferroni correction, our results indicated that DS patients had more higher CRP levels than NDS patients (Z = 6.003, Bonferroni corrected P < 0.001) and HCs (Z = 4.942, Bonferroni corrected P < 0.001), and NDS patients had higher levels of IL-1β (Z = 2.635, Bonferroni corrected P = 0.025), IFN-γ (Z = 3.007, Bonferroni corrected P = 0.008), and proinflammatory cytokines (Z = 2.630, Bonferroni corrected P = 0.026) than HCs. In addition, the levels of IL-1β (Z = 3.067, Bonferroni corrected P = 0.006), IL-6 (Z = 3.902, Bonferroni corrected P < 0.001), IL8 (Z = 3.595, Bonferroni corrected P = 0.001), IFN-γ (Z = 3.052, Bonferroni corrected P = 0.007), proinflammatory cytokines (Z = 3.477, Bonferroni corrected P = 0.002) in DS patients were higher than that those in HCs. Data in the **Table 2** are presented as the median with interquartile range. Additionally, sex stratification analysis revealed that all cytokines except IL-12 were significantly different among three female groups, but there were no statistical differences in all cytokines in male groups (**Table S1** and **Figure S1** in the **Supplementary Materials**).

**Table 2:** Plasma proteins among control subjects, and deficit and nondeficit schizophrenia patients.

	DS (N = 51)	NDS (N = 90)	HCs (N = 67)	Z	P
CRP (pg/ml)	13.20 ± 10.97	6.85 ± 2.72	6.97 ± 2.60	38.802	< 0.001*
IL-1β (pg/ml)	20.79 ± 20.21	20.02 ± 10.94	15.90 ± 9.89	11.042	0.004*
Il-2 (pg/ml)	219.70 ± 211.34	195.89 ± 89.10	187.99 ± 78.46	5.409	0.067
IL-4 (pg/ml)	66.99 ± 47.53	67.56 ± 34.22	70.35 ± 27.13	3.778	0.151
IL-6 (pg/ml)	87.83 ± 52.95	79.79 ± 34.66	74.62 ± 25.35	15.311	< 0.001*
IL-8 (pg/ml)	127.05 ± 104.00	109.68 ± 55.92	100.56 ± 56.50	12.997	0.002*
IL-10 (pg/ml)	31.84 ± 28.64	37.64 ± 20.41	36.69 ± 17.04	3.627	0.163
IL-12 (pg/ml)	26.72 ± 30.01	24.78 ± 17.50	26.03 ± 12.04	0.808	0.668
IL-17 (pg/ml)	25.12 ± 17.92	21.54 ± 12.35	22.73 ± 10.89	4.914	0.086
TNF-α (pg/ml)	57.69 ± 47.10	52.77 ± 24.26	51.74 ± 22.50	5.035	0.081
IFN-γ (pg/ml)	509.99 ± 392.25	479.39 ± 187.23	411.11 ± 157.01	12.259	0.002*
Pro (pg/ml)	965.04 ± 980.36	948.47 ± 268.81	885.52 ± 156.58	13.167	0.001*
Anti (pg/ml)	105.77 ± 74.48	109.49 ± 36.12	108.50 ± 30.68	3.637	0.162

Pro = proinflammatory cytokines; Anti = anti-inflammatory cytokines. Data were presented in Media ± interquartile. *P < 0.05.

### Correlation of cytokines and clinical symptoms in schizophrenia patients

**Figure 2:**
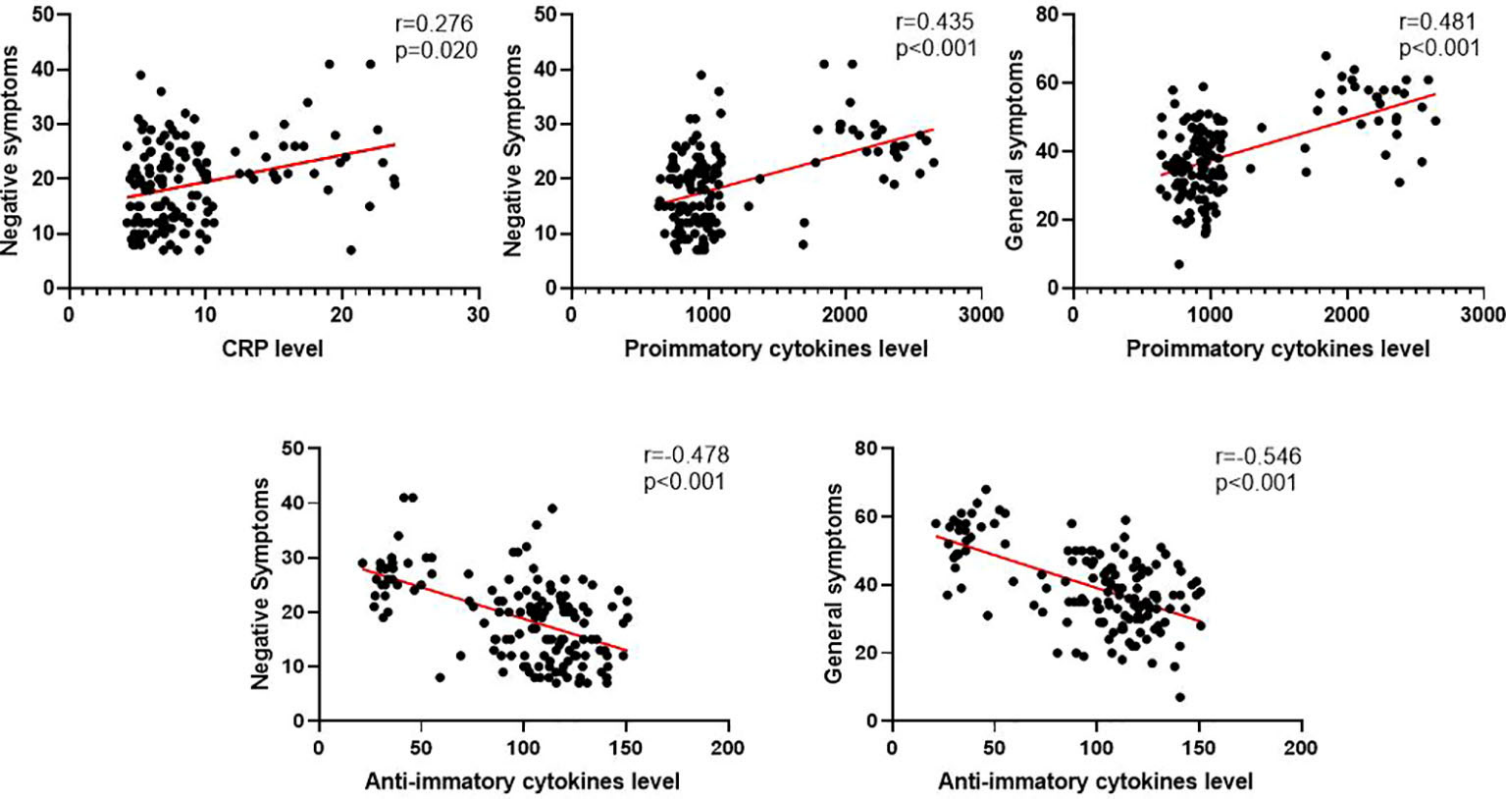
Significant correlation between cytokines and various clinical symptoms in SCZ patients.

After controlling for factors such as sex, age, BMI, education level, age of onset, total disease course, family history, and medical history data, our results indicated that plasma CRP levels were positively correlated with negative symptoms (r = 0.276, P = 0.020). Furthermore, negative symptoms, general psychiatric symptoms, and PANSS total score were positively correlated with proinflammatory cytokines (r = 0.435, P < 0.001; r = 0.481, P < 0.001; r = 0.442, P < 0.001). Negative symptoms, general psychiatric symptoms, and the PANSS total score were negatively correlated with anti-inflammatory cytokines (r = -0.478, < 0.001; r = -0.546, P < 0.001; r = -0.524, P < 0.001) (see in **Figure 2**). The correlations between cytokines and clinical symptoms after sex stratification in schizophrenia patients are shown in **Table S2** (see in **Supplementary Materials**).

### Risk factors of DS patients

As shown in **Table S3** (see **Supplementary Materials**), univariate logistic regression analysis demonstrated that education, CRP, IL-2, IL-6, IL-8, and TNF-α might determine whether the patient has DS. The results of further incorporating the above factors (P < 0.05) into the stepwise logistic regression analysis revealed that CRP is an independent risk factor for DS (B = -0.448, SE = 0.090, Wald = 24.882, OR = 0.639, P < 0.001). Our results showed that CRP had a good performance to classify DS and NDS (ROC-AUC = 79.3% (70.1%-88.5%)), resulting in an optimal sensitivity of 94.4% and an optimal specificity of 66.7% (See in **Figure S2**).

## Discussion

The main findings of the present study are as follows: 1) DS patients accounted for 36.17% of the total number of schizophrenia patients; 2) both DS and NDS patients had poorer cognitive function than HC group, especially DS patients, who showed more severe cognitive impairment than NDS patients in the immediate memory, attention, and language dimensions of RBANS; 3) altered immune-inflammatory components were found in schizophrenia, but with different immune-inflammatory characteristics between DS and NDS patients when compared to the HC group; 4) the immune-inflammatory characteristics between DS and NDS patients may have sex differences.

The proportion of DS patients reported in our study (36.17%) was higher than that found in some previous studies that used the Schedule for Deficit Syndrome (SDS) for categorizing deficit syndrome (25, 26), but was similar to some others that used the PDS scale (4, 27). Interestingly, a recent meta-analysis summarized the existing research with the SDS scale and calculated the pooled proportion of the DS subgroup was 32.64% (28). Based on the present study, up to one-third of patients with schizophrenia might have enduring and primary negative symptoms that lack effective treatment to date. The inconsistency in the prevalence of DS in schizophrenia may be largely caused by the different evaluation tools used. To the best of our knowledge, many evaluation tools could be used to detect persistent negative symptoms, such as the PDS, the SDS, the Scale for the Assessment of Negative Symptoms (SANS), the negative factor of the PANSS, or the negative symptom subscale of the Brief Psychiatric Rating Scale (BPRS). Furthermore, the different disease courses of schizophrenia may also be a major cause of this inconsistency, as a recent study demonstrated that the prevalence of DS among Chinese patients with first-episode schizophrenia was 23.0% (8), and longitudinal studies indicated that the prevalence of DS was unstable during treatment (29). Taken together, future studies are warranted to accurately identify schizophrenia patients with deficit syndrome with a higher quality research design to control for confounding factors.

Cognitive impairment is also a core symptom of schizophrenia; it occurs in the early stage of schizophrenia patients and usually persists at all stages of this disorder. Our previous work indicated a wide range of cognitive impairment in first-episode and drug-naïve schizophrenia patients (30). In the present study, we further found that both DS and NDS patients with a total disease course over one year also had extensive and severe cognitive impairment compared to the HC group. Previous studies also demonstrated that cognitive function was more seriously impaired in chronic schizophrenia patients than in first-episode drug-naïve patients (31). This evidence supports that cognitive deficits exist in the early onset of schizophrenia patients and deteriorate over the chronic progressive course. Interestingly, our present study indicated that DS patients have greater cognitive impairment than NDS patients, especially in the immediate memory, attention, and language dimensions of cognition evaluated by RBANS, which is in line with the findings of previous studies (8, 10). Negative symptoms in schizophrenia patients consistently aggravate cognitive impairment and further lead to a worse level of psychosocial functioning (24, 32). A recent 10-year follow-up study reported that schizophrenia patients with sustained negative symptoms could have a poorer cognitive function and a lower level of global functioning compared to patients with an early illness course characterized by the absence of negative symptoms (33). In addition, transcranial direct current stimulation (tDCS) or repetitive transcranial magnetic stimulation (rTMS) can ameliorate the negative symptoms and further improve cognitive function in schizophrenia patients (34, 35). The greater extent of cognitive deficits among DS patients than among NDS patients found in our present study further supports the above causal relationship. However, the exact mechanisms by which negative symptoms may deteriorate cognitive function in schizophrenia patients are still unknown. Whether the negative symptoms and cognitive deficits share the common brain and biological mechanisms warrants more exploration.

Existing evidence indicates that maternal infection during pregnancy is implicated with an increased risk of schizophrenia in the offspring (36). Therefore, it has been proposed that immunologic abnormalities are likely to be involved in the development of schizophrenia (37), and several previous studies did find the upregulation of proinflammatory cytokine levels and downregulation of anti-inflammatory cytokine levels in first-episode and drug-naïve schizophrenia patients (38, 39), while some other studies failed to replicate the results and even presented the opposite direction (40, 41). A recent study found that antipsychotic drugs could modulate inflammation levels and alleviate clinical symptoms in schizophrenia (42), but also with mixed results between other studies (43). The discrepancies in studies may be attributed to several factors, including different sample sizes, antipsychotic drugs used, duration of treatment, and different disease statuses. Interestingly, although short-term olanzapine treatment exhibited significant improvements in cognitive function in schizophrenia patients (44), previous studies found that long-term clinical use of olanzapine causes adverse metabolic effects, including weight gain and alterations in lipid and glucose metabolism, which are associated with a state of chronic and low-grade inflammation, and consequently reduce levels of brain-derived neurotrophic factor, resulting in neurological damage and secondary cognitive impairment (45, 46). Hence, the discrepancies between studies may be related to the antipsychotics used and the duration of treatment. More importantly, the high heterogeneity of schizophrenia patients may first contribute to this inconclusiveness between studies. Clinically, schizophrenia is a heterogeneous disease that manifests a variety of symptoms, which poses challenges to understanding its neurobiological mechanisms (47). In turn, classifying different subtypes and subpopulations of the disorder was regarded as a productive approach to overcoming this heterogeneity (27). An increasing amount of evidence has shown that DS is a homogeneous subtype of schizophrenia, with unique pathophysiological and psychological features. For instance, Bryant et al. reported a significant difference in white matter neurometabolic signatures between DS and NDS patients using single-voxel magnetic resonance spectroscopy technology (48). Zhang et al. found that DS patients had different eye movement characteristics than NDS patients (49). Moreover, some previous studies also provided evidence suggesting an association between the DS subtype of schizophrenia and gene polymorphisms, including dopamine D2 receptor and catechol-O-methyltransferase (COMT) genes (50, 51). Despite these advances, more potential biological differences need to be explored to deepen our understanding of its biomechanism and further promote the development of therapeutics.

The immune system may also be involved in this distinct neurobiology. In the present study, we found abnormal inflammatory cytokines among the DS, NDS, and HC groups. Both the DS and NDS patients showed significantly higher levels of INF-γ and total proinflammatory cytokines than the HC group. Elevated levels of INF-γ have been repeatedly reported in first-episode drug-naïve schizophrenia (38) and chronic schizophrenia (52). Interestingly, our results showed that DS patients had a significantly increased CRP level compared to NDS patients and HCs, which was consistent with previous work demonstrating an elevated CRP level in newly-diagnosed drug-naïve patients with DS (17). Two past studies found higher concentrations of IL-6 in DS patients than in the NDS and HC groups (17, 53), but we here only found a significant difference in IL-6 between DS and HC groups. Even so, all these findings support the notion that deficit schizophrenia may represent a distinct subtype with a specific immune inflammatory mechanism. Since limited research focuses on this subtype of schizophrenia, further studies are required to verify the existing findings.

Interestingly, our sex stratification analysis found that the differences in immune inflammation among DS, NDS and HC groups were mainly observed in female samples and involved almost all examined cytokines. It appears that DS patients has higher levels of inflammatory activation than NDS patients only in females. We know ample evidence supports significant sex differences in many aspects of schizophrenia, including clinical symptoms, cognitive impairments, and biological mechanisms (54–56). One plausible explanation for the sex differences in immune inflammation between DS and NDS patients has to do with sex hormones. It is known that menopause and ovariectomy can generate a low grade of systemic inflammation, and Estrogen and progesterone have a regulatory effect on immune inflammation (57). These physical differences may cause sex difference in the relationship between immune-inflammatory and DS. However, no studies to date investigate the sex differences in immune characteristics in DS and NDS patients, our preliminary findings with a small sex-stratified sample should be interpreted with caution. Future studies with larger samples are warrant to verify our findings and to deeply understanding its biological mechanisms.

There are several limitations in the present study. First, its cross-sectional design makes it impossible to draw any conclusions and involved a wider range of cytokines about the directionality of the association between inflammation and negative symptoms in schizophrenia patients. Second, although we recorded the antipsychotics that the patient was currently taking and compared the equal effective dose of olanzapine between groups, we did not record or consider the impact of past antipsychotic use on the results. Hence, future longitudinal studies are warranted to verify our findings and consider the drug effects. Third, the sample size after the sex stratification analysis is relatively small, our stratified results should be verified in future larger studies. Fourth, the SDS scale may be a better choice for categorizing DS patients than the PDS. Fifth, the patients were all outpatients recruited from one hospital in Shanghai in this survey, and the findings could not be generalized to other settings or inpatients. Therefore, the findings in the present study should be replicated in other studies using more standard assessment tools before more generalized conclusions can be drawn.

## Conclusions

In summary, our results indicated that DS patients had greater cognitive impairment than the NDS and HC groups. Moreover, DS patients showed different characteristics of immune inflammation and even higher levels of CRP and proinflammatory cytokines than NDS patients, especially in females. These findings suggest that DS patients are a major homogenous subtype of schizophrenia and support the hypothesis of a separate disease of DS. Future studies could potentially benefit from stratifying patients accordingly when investigating the efficacy of novel pharmacotherapies.

## Data availability statement

The original contributions presented in the study are included in the article/**Supplementary Material**. Further inquiries can be directed to the corresponding authors.

## Ethics statement

The studies involving human participants were reviewed and approved by Medical Ethics Committee of Shanghai Mental Health Center. The patients/participants provided their written informed consent to participate in this study.

## Author contributions

DW and CZ contributed to the overall design of the study. DW, XF and CZ wrote the protocol for the genotyping. YW, YC, LY, ZW, RL and JR collected the included samples. DW and CZ undertook the statistical analysis and interpretation of the data. DW and XF wrote the manuscript. All authors contributed to the article and approved the submitted version.

## Funding

This work was supported by the National Key Research and Development Program of China (2018YFC1314302), National Natural Science Foundation of China (81471358 and 81771450), Western Medicine Guide Project of Shanghai Municipal Commission of Science and Technology (14411969000), Key Project supported by the Medical Science and Technology Development Foundation, Nanjing Department of Health (YKK20090), and Science and Technology Development Program of Nanjing Medical University (NMUB2019107).

## Acknowledgments

We are deeply grateful to all participants.

## Conflict of interest

The authors declare that the research was conducted in the absence of any commercial or financial relationships that could be construed as a potential conflict of interest.

## Publisher’s note

All claims expressed in this article are solely those of the authors and do not necessarily represent those of their affiliated organizations, or those of the publisher, the editors and the reviewers. Any product that may be evaluated in this article, or claim that may be made by its manufacturer, is not guaranteed or endorsed by the publisher.
