# Supplementary material for: Differences in inflammatory marker profiles and cognitive functioning between deficit and nondeficit schizophrenia
Source: Front Immunol. 2022 Oct 19;13:958972. doi: 10.3389/fimmu.2022.958972 (PMC9627304; doi:10.3389/fimmu.2022.958972)
Supplement: Supplementary file 1 [file DataSheet_1.docx]

|  |  | DS (N=51) | NDS (N=90) | HCs (N=67) | Z | P |
| --- | --- | --- | --- | --- | --- | --- |
| CRP (pg/ml) | Male | 10.04 ± 11.59 | 7.21 ± 3.76 | 7.59 ± 2.47 | 4.350 | 0.114 |
|  | Female | 14.96 ± 9.14 | 6.49 ± 2.64 | 6.34 ± 2.51 | 39.057 | < 0.001 |
| IL-1β (pg/ml) | Male | 16.70 ± 11.25 | 18.16 ± 11.22 | 18.35 ± 11.22 | 0.535 | 0.765 |
|  | Female | 24.43 ± 32.08 | 20.77 ± 11.15 | 14.40 ± 7.37 | 20.983 | 0.000 |
| Il-2 (pg/ml) | Male | 194.99 ± 87.69 | 194.68 ± 70.71 | 183.70 ± 65.56 | 0.561 | 0.755 |
|  | Female | 237.51± 288.14 | 198.87 ± 99.04 | 192.60 ± 86.88 | 7.258 | 0.027 |
| IL-4 (pg/ml) | Male | 71.55 ± 19.46 | 70.33 ± 33.45 | 67.49 ± 28.53 | 0.021 | 0.989 |
|  | Female | 56.08 ± 55.79 | 66.51 ± 33.79 | 72.85 ± 26.47 | 7.756 | 0.021 |
| IL-6 (pg/ml) | Male | 84.36 ± 30.88 | 80.29 ± 32.47 | 78.28 ± 29.14 | 2.137 | 0.344 |
|  | Female | 100.87 ± 79.95 | 78.50 ± 39.92 | 73.26 ± 26.98 | 16.557 | < 0.001 |
| IL-8 (pg/ml) | Male | 108.87 ± 33.54 | 100.93 ± 49.48 | 106.50 ± 60.72 | 1.449 | 0.485 |
|  | Female | 135.47 ± 169.01 | 123.06 ± 56.72 | 97.41 ± 52.23 | 18.386 | < 0.001 |
| IL-10 (pg/ml) | Male | 35.77 ± 19.31 | 36.55 ± 16.33 | 35.60 ± 12.28 | 0.229 | 0.892 |
|  | Female | 22.16 ± 31.78 | 39.16 ± 22.36 | 39.37 ± 19.39 | 7.891 | 0.019 |
| IL-12 (pg/ml) | Male | 22.17 ± 13.06 | 25.83 ± 13.94 | 28.73 ± 11.43 | 0.955 | 0.620 |
|  | Female | 29.89 ± 48.05 | 23.86 ± 19.01 | 25.67 ± 13.03 | 4.264 | 0.119 |
| IL-17 (pg/ml) | Male | 22.35 ± 11.10 | 20.67 ± 14.24 | 23.93 ± 7.29 | 0.640 | 0.726 |
|  | Female | 27.82 ± 30.13 | 21.63 ± 11.78 | 20.86 ± 11.55 | 9.786 | 0.008 |
| TNF-α (pg/ml) | Male | 51.33 ± 23.88 | 50.94 ± 22.35 | 51.55 ± 24.68 | 0.581 | 0.748 |
|  | Female | 64.90 ± 83.14 | 53.59 ± 25.56 | 51.74 ± 21.35 | 7.382 | 0.025 |
| IFN-γ (pg/ml) | Male | 464.20 ± 133.88 | 449.95 ± 212.97 | 411.59 ±150.99 | 1.832 | 0.400 |
|  | Female | 541.89 ± 620.23 | 488.04 ± 147.83 | 411.11 ± 179.22 | 11.356 | 0.003 |
| Pro (pg/ml) | Male | 948.20 ± 191.30 | 923.64 ± 223.73 | 890.71 ± 126.11 | 2.192 | 0.334 |
|  | Female | 1023.88 ± 1378.08 | 969.77 ± 256.08 | 873.68 ± 168.81 | 12.982 | 0.002 |
| Anti (pg/ml) | Male | 106.61 ± 28.36 | 107.91 ± 34.04 | 104.34 ± 30.08 | 0.029 | 0.986 |
|  | Female | 101.41 ±83.78 | 110.54 ± 37.29 | 109.34 ± 29.66 | 7.534 | 0.023 |

Table S1. Plasma proteins among control subjects, and deficit and nondeficit schizophrenia patients after sex stratification

Table S2. Results of partial correlation analysis between cytokines and various clinical symptoms in SCZ patients of different sex

|  |  | Positive subscale | | Negative subscale | | General subscale | | PANSS Total score | |
| --- | --- | --- | --- | --- | --- | --- | --- | --- | --- |
|  |  | r | P | r | P | r | P | r | P |
| CRP(mg/L) | M | -0.204 | 0.350 | -0.003 | 0.988 | -0.160 | 0.466 | -0.175 | 0.425 |
|  | F | -0.088 | 0.582 | 0.372 | 0.015 | 0.219 | 0.164 | 0.229 | 0.145 |
| IL-1β(pg/mL) | M | 0.389 | 0.066 | 0.290 | 0.179 | 0.474 | 0.022 | 0.445 | 0.033 |
|  | F | 0.044 | 0.784 | 0.422 | 0.005 | 0.457 | 0.002 | 0.407 | 0.007 |
| IL-2(pg/mL) | M | 0.271 | 0.211 | 0.331 | 0.123 | 0.434 | 0.039 | 0.401 | 0.058 |
|  | F | 0.057 | 0.721 | 0.545 | <0.001 | 0.520 | <0.001 | 0.490 | 0.001 |
| IL-4(pg/mL) | M | -0.449 | 0.031 | -0.551 | 0.006 | -0.602 | 0.002 | -0.636 | 0.001 |
|  | F | 0.016 | 0.918 | -0.380 | 0.013 | -0.420 | 0.006 | -0.356 | 0.021 |
| IL-6(pg/mL) | M | 0.332 | 0.121 | 0.433 | 0.039 | 0.474 | 0.022 | 0.501 | 0.015 |
|  | F | 0.048 | 0.764 | 0.453 | 0.003 | 0.468 | 0.002 | 0.426 | 0.005 |
| IL-8(pg/mL) | M | 0.418 | 0.047 | 0.519 | 0.011 | 0.587 | 0.003 | 0.600 | 0.002 |
|  | F | -0.099 | 0.533 | 0.486 | 0.001 | 0.396 | 0.009 | 0.360 | 0.019 |
| IL-10(pg/mL) | M | -0.583 | 0.004 | -0.296 | 0.170 | -0.492 | 0.017 | -0.546 | 0.007 |
|  | F | -0.104 | 0.514 | -0.556 | <0.001 | -0.577 | <0.001 | -0.537 | <0.001 |
| IL-12(pg/mL) | M | 0.379 | 0.074 | 0.262 | 0.226 | 0.381 | 0.073 | 0.396 | 0.061 |
|  | F | 0.125 | 0.429 | 0.498 | 0.001 | 0.544 | <0.001 | 0.504 | 0.001 |
| IL-17(pg/mL) | M | 0.256 | 0.239 | 0.443 | 0.034 | 0.453 | 0.03 | 0.451 | 0.031 |
|  | F | 0.216 | 0.170 | 0.520 | <0.001 | 0.627 | <0.001 | 0.580 | <0.001 |
| TNF-α(pg/mL) | M | 0.387 | 0.068 | 0.405 | 0.055 | 0.561 | 0.005 | 0.549 | 0.007 |
|  | F | -0.050 | 0.754 | 0.500 | 0.001 | 0.470 | 0.002 | 0.417 | 0.006 |
| IFN-γ(pg/mL) | M | 0.275 | 0.204 | 0.415 | 0.049 | 0.512 | 0.012 | 0.489 | 0.018 |
|  | F | 0.101 | 0.526 | 0.321 | 0.038 | 0.399 | 0.009 | 0.355 | 0.021 |
| Proinflammatory | M | 0.346 | 0.106 | 0.454 | 0.03 | 0.562 | 0.005 | 0.544 | 0.007 |
|  | F | 0.066 | 0.679 | 0.457 | 0.002 | 0.485 | 0.001 | 0.441 | 0.003 |
| Anti-inflammatory | M | -0.544 | 0.007 | -0.510 | 0.013 | -0.621 | 0.002 | -0.666 | 0.001 |
|  | F | -0.029 | 0.856 | -0.487 | 0.001 | -0.524 | <0.001 | -0.462 | 0.002 |

Table S3. Results of univariate Logistic regression analysis of all factors in deficit and non-deficit schizophrenia patients

| Factors | BE | SE | Wald | Sig | Exp(B) | EXP(B)95% CI | |
| --- | --- | --- | --- | --- | --- | --- | --- |
|  |  |  |  |  |  | Lower | Upper |
| Age | 0.024 | 0.019 | 1.535 | 0.215 | 1.024 | 0.986 | 1.064 |
| Sex | -0.381 | 0.355 | 1.154 | 0.283 | 0.683 | 0.341 | 1.369 |
| BMI | -0.014 | 0.050 | 0.081 | 0.776 | 0.986 | 0.893 | 1.088 |
| Education | 0.127 | 0.062 | 4.192 | 0.041 | 1.135 | 1.005 | 1.281 |
| Age of onset | -0.001 | 0.023 | 0.001 | 0.973 | 0.999 | 0.956 | 1.045 |
| Total disease course | 0.061 | 0.049 | 1.506 | 0.220 | 1.062 | 0.964 | 1.170 |
| Family history | 0.056 | 0.404 | 0.019 | 0.890 | 1.057 | 0.479 | 2.335 |
| Chlorpromazine Equivalents | -0.002 | 0.001 | 2．028 | 0.154 | 0.998 | 0.995 | 1.001 |
| **PANSS** |  |  |  |  |  |  |  |
| Positive subscale | 0.104 | 0.030 | 11.886 | 0.001 | 1.109 | 1.046 | 1.177 |
| Negative subscale | -0.111 | 0.027 | 16.650 | 0.000 | 0.894 | 0.848 | 0.944 |
| General subscale | 0.011 | 0.015 | 0.573 | 0.449 | 1.012 | 0.982 | 1.042 |
| Total score | 0.000 | 0.008 | 0.001 | 0.973 | 1.000 | 0.985 | 1.015 |
| **RBANS** |  |  |  |  |  |  |  |
| Immediate memory | 0.028 | 0.011 | 6.463 | 0.011 | 1.029 | 1.007 | 1.051 |
| Visuospatial constructional | 0.006 | 0.011 | 0.297 | 0.586 | 1.006 | 0.984 | 1.028 |
| Attention | 0.021 | 0.011 | 3.918 | 0.048 | 1.022 | 1.000 | 1.044 |
| Language | 0.027 | 0.012 | 5.006 | 0.025 | 1.028 | 1.003 | 1.053 |
| Delayed memory | 0.014 | 0.009 | 2.187 | 0.139 | 1.014 | 0.995 | 1.033 |
| Total score | 0.031 | 0.031 | 5.922 | 0.015 | 1.031 | 1.006 | 1.057 |
| CRP | -0.413 | 0.080 | 26.462 | 0.000 | 0.661 | 0.565 | 0.774 |
| IL-1β | -0.015 | 0.012 | 1.560 | 0.212 | 0.985 | 0.963 | 1.008 |
| IL-2 | -0.003 | 0.001 | 3.702 | 0.054 | 0.997 | 0.994 | 1.000 |
| IL-4 | 0.010 | 0.007 | 1.948 | 0.163 | 1.010 | 0.996 | 1.025 |
| IL-6 | -0.007 | 0.004 | 2.919 | 0.088 | 0.993 | 0.985 | 1.001 |
| IL-8 | -0.006 | 0.003 | 5.531 | 0.019 | 0.994 | 0.989 | 0.999 |
| IL-10 | 0.018 | 0.012 | 2.265 | 0.132 | 1.018 | 0.994 | 1.043 |
| IL-12 | -0.010 | 0.009 | 1.325 | 0.250 | 0.990 | 0.973 | 1.007 |
| IL-17 | -0.015 | 0.011 | 1.921 | 0.166 | 0.985 | 0.965 | 1.006 |
| TNF-α | -0.009 | 0.005 | 2.996 | 0.083 | 0.991 | 0.981 | 1.001 |
| IFN-γ | 0.000 | 0.001 | 0.241 | 0.624 | 1.000 | 0.998 | 1.001 |
| Pro | 0.000 | 0.000 | 1.811 | 0.178 | 1.000 | 0.999 | 1.000 |
| Anti | 0.008 | 0.005 | 2.520 | 0.112 | 1.008 | 0.998 | 1.018 |

Fig. S1. Plasma levels of inflammatory cytokines in control subjects, deficit and nondeficit schizophrenia patients in different sex.


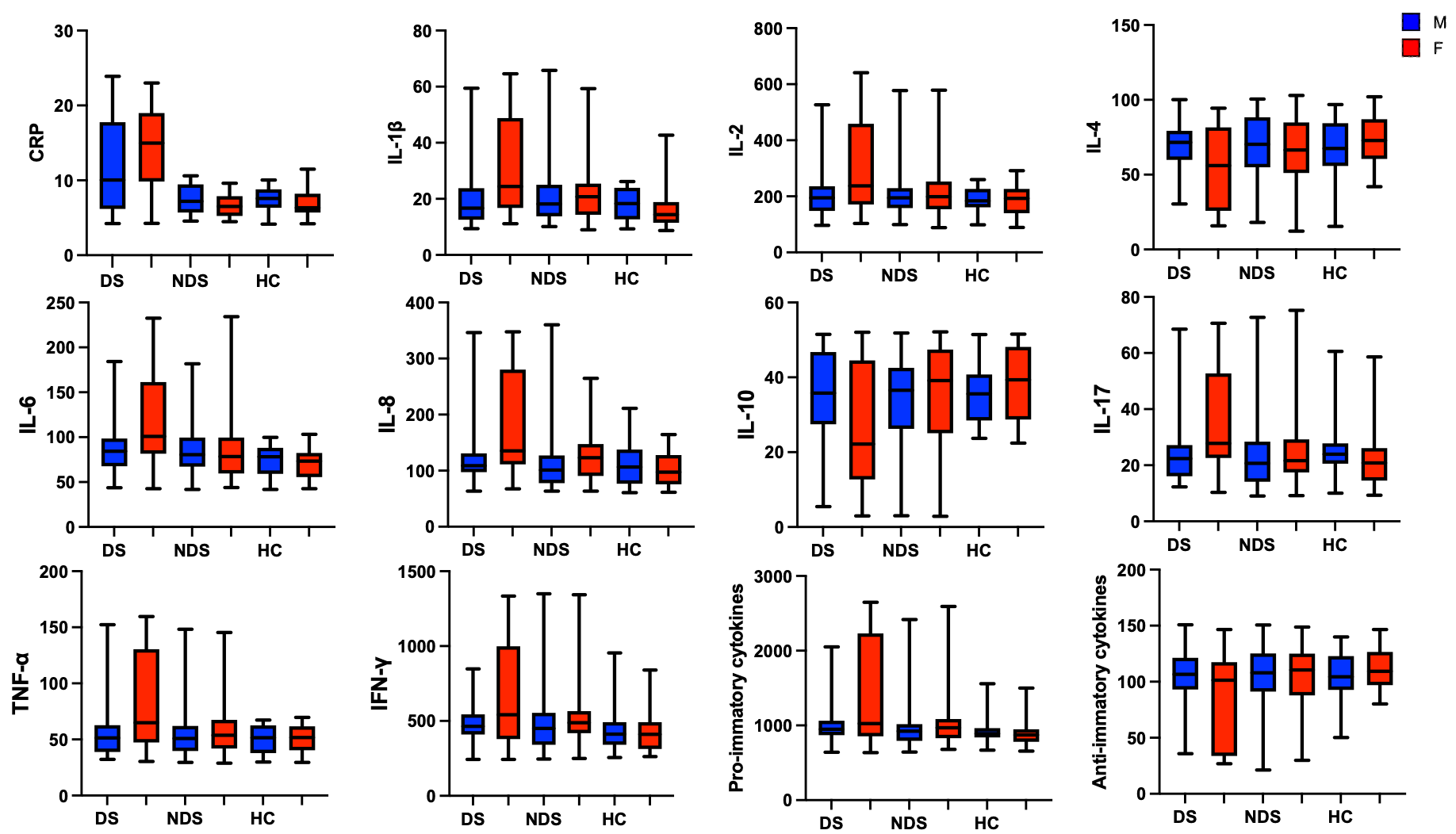


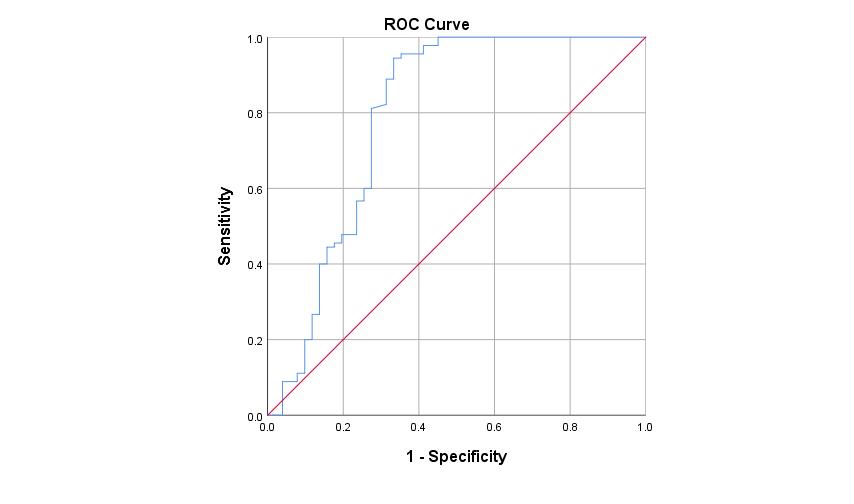
Fig. S2 Receiver operating characteristic (ROC) curves of the optimal sensitivity and specificity by using CRP level to differentiate deficit and nondeficit schizophrenia patients.
